# Examining Factors Associated with the Use of Community Food Resources: An Application of the Andersen Model to Inform Future Interventions

**DOI:** 10.3390/ijerph21010076

**Published:** 2024-01-09

**Authors:** Abiodun T. Atoloye, Oluyemisi Akinsola, Melissa Murillo

**Affiliations:** Department of Nutrition, Dietetics, and Food Sciences, Utah State University, Logan, UT 84322, USA

**Keywords:** community food resources, family composition, racial and ethnic diversity, food environment, food access, mobility, mobile food services, food security, Andersen Model

## Abstract

The role of the food environment in shaping nutrition and health has gained substantial attention from policymakers, public health researchers, and advocacy groups. To promote equities in food access and nutrition outcomes, understanding factors linked with the utilization of local community food resources is crucial. Using Andersen’s service utilization model, we explained how adults use their neighborhood food resources. In a cross-sectional study design, an online survey was conducted in REDCap Version 13.4.0 via the Amazon Mechanical Turk (MTurk) involving 1830 adults with a mean age of 37.9  ±  12.1 years. Participants answered questions on predisposing, enabling, and need factors that influence their use of different community food resources. The predisposing factors that were statistically significant included age, family size, marital status, race, and ethnicity. The enabling factors included travel time, travel mode, income, and shopping decision motivators (such as being able to use Special Supplemental Nutrition Program for Women, Infants, and Children (WIC) vouchers, delivery services, great sales, and coupons). Food security and community food resources need for lower food price were the significant need factors. However, these factors vary by the types of food resources. In conclusion, enhancing the utilization of community-based food access initiatives and programs among underserved families requires consideration of family composition, racial and ethnic diversity, and transportation access.

## 1. Introduction

Differential access to resources and amenities is associated with disparities in health outcomes [1]. The differences observed cannot be entirely explained by the characteristics of individuals living in those neighborhoods but include other contextual factors, such as structural and systemic inequalities [1]. The food environment has become a critical determinant of nutrition and health status, which has attracted attention from policymakers, public health researchers, and advocacy groups. Amid ongoing efforts to tackle food access challenges and related health disparities, local communities and residents often develop their own unique methods for accessing and consuming healthy diets [2,3]. Recent food environment research has highlighted shifts in food retail trends, with dollar stores and corner stores now serving as significant sources of food security for disadvantaged and rural communities [4]. However, there remains a limited understanding of the factors that influence the utilization of different community food resources.

To promote equity in food access and nutrition-related outcomes, understanding the factors that shape the utilization of local community food resources is paramount. We applied Andersen’s service utilization model [5] to elucidate on the usage of neighborhood food resources among American adults. The model proposes that the use of a particular resource or service is influenced by predisposing, enabling, and need factors [5]. Originally developed for healthcare service research, this model has been widely employed to identify healthcare access and utilization disparities among diverse populations. Predisposing factors encompass an individual’s inclination to use healthcare services, enabling factors involve an individual’s ability to access services, and need factors relate to an individual’s health status and necessity for services [5]. By considering these factors, healthcare providers and policymakers have identified population groups facing barriers to care access and design targeted interventions to mitigate these disparities [6]. However, only a handful of studies have applied a similar approach to investigate food access or food security within larger American populations. For instance, Lun (2004) [7] utilized Andersen’s model to examine how the elderly engage with community-based services such as meal delivery and congregate meals. Other applications include investigating the use of informal food support among low-income households [8] and exploring factors influencing Supplemental Nutrition Assistance Program (SNAP) benefit utilization [9]. Furthermore, Sharareh and Wallace (2022) [10] employed the model to highlight the role of nonprofit organizations in comprehending and addressing food insecurity and associated social needs like housing and healthcare issues. By employing the Andersen model in the context of community food resources, we can gain insights into factors impacting food access and develop targeted strategies to improve food security and nutrition outcomes.

While these previous studies seem to focus on charitable and/or federally funded food support resources and food security, this current study examines the contextual factors associated with using different community food resource types. We define the relevant factors as follows: (a) the predisposing factors distinguish traditionally vulnerable groups to access some types of food; (b) enabling factors facilitate or impede access to healthy food; and (c) need factors are expected to have a substantial bearing on access to food.

## 2. Materials and Methods

This study is based on a cross-sectional analysis of a survey programmed in REDCap (a web-based application designed to support data capture for research studies) [11] and administered via Amazon Mechanical Turk (MTurk). MTurk is a crowdsourcing website widely used to obtain quality data rapidly and inexpensively [12]. A multi-item sample questionnaire was first developed based on the comprehensive literature on food access and diet to ensure face validity [13,14,15,16]. The draft questionnaire was sent to public health experts on food security and access (*n* = 3) to check for construct and content validity. The expert suggestions were incorporated. Furthermore, think-aloud cognitive interviews were conducted with food shoppers (*n* = 2) to ensure content validity [17]. The interviews tested all survey questions, emphasizing the pretest of the food store access, and shopping habits questions. The interviews checked if respondents understood the questions correctly and could provide accurate answers. Each cognitive testing session lasted 50–60 minutes. Two other individuals pretested the survey to check if respondents could complete it within a reasonable time. Question wording and overall flow were modified in the final questionnaire based on feedback from both groups. However, their responses to the questionnaire were not included as data for this analysis.

The study sample size was estimated as 1600 based on the total population of adults in the US (*n*  ≈  333 million), with 99% confidence levels and a conservative 3% margin of error [18]. The eligibility criteria for the study were programmed into the survey administration platform, MTurk. Participants had to be 18 years or older, reside in the United States, and be able to read and respond in English. After reading the study information page, participants consented to participate by clicking the REDCap survey hyperlink in MTurk. The participants then completed a 20 minutes survey in REDCap. Data were collected from the 13th of February to 22nd of March, 2023.

To enhance data quality in an online survey, researchers screened for participants who clicked the study hyperlink but did not complete at least one full survey page and displayed inattentiveness as evidenced by providing incoherent answers to some pre-selected “red herring” questions. The “red herring” questions included non-US states and counties matching the Zip code reported and birthplace. A total of 5997 individuals clicked on the study hyperlink that took them to the study landing page in REDCap; 4099 (68.3%) were dropped for inattentiveness or incompleteness. Study respondents (*n* = 1898, 31.6%) received USD 1.20 for their time; this dollar value is within the acceptable incentive for a survey conducted on Amazon MTurk [19,20]. An additional 68 individuals (*n* = 1.1%) were dropped during data cleaning for incoherent responses to the number of children or adults in the home, and 1830 (30.5%) were finally included in the current analysis. The institutional review board at Utah State University approved all study procedures on an expedited review.

### 2.1. Measures

To initiate the Anderson Behavior Model, the following blocks of information were collected from the study participants.

For the predisposing factors, participants were asked about their age (in years), and the number of adults, older children, and younger children in their homes, and these were treated as continuous variables. Participants responded “yes” or “no” to being Hispanic (ethnicity) and the race variable options were White/Caucasians, Blacks, Asians, Native Hawaiians, and American Indians. Gender included response options such female, male, transgender woman, transgender man, prefer not to answer, and decline.

The enabling factors included the total annual income variable ranging from less than USD 25,000 to above USD 150,000, and travel time (in minutes) from home-to-store for food shopping. The motivators of shopping decisions encompassed four variables related to factors influencing the choice of the store where respondents predominantly purchase their food. These variables included great sales and coupons, low food prices, the use of WIC or SNAP/Double Up Food Bucks, and the utilization of food delivery services. Participants were asked to rate the importance of each factor on a Likert scale: from “not at all important” to “very important” plus a “not applicable” option. The shopping travel mode considers the store where participants primarily buy their food and their usual transportation mode to the store with a “check all that apply options”. The shopping travel options provided include walk, bus, bicycle, public transportation, private car, neighbor’s car, friend’s car, paid ride, and home delivery.

For the need factors, food security information was collected using the 2-item hunger vital sign screening questionnaire [21]. The questions included statements such as (i) “Within the past 12 months, I/we were worried whether our food would run out before we got money to buy more” and (ii) “Within the past 12 months, the food I/we bought just didn’t last, and I/we didn’t have the money to get more. Response options “often true”, “sometimes true”, and “never true” were provided. Based on responses to the two questions, a food security score was estimated for each participant (affirmative responses “sometimes true” and “often true” were coded as 1 and “never true” as 0 and summed for both questions). Food security levels were then created from the food security scores with a score of 0 as food secure and scores of 1 and 2 indicated food insecure. Additionally, perceived community-level food-related needs were assessed using 11 variables. These variables encompass factors such as improved food selection, a wide variety of foods, culturally relevant foods, lower food prices, better food quality, diverse food options, convenient access to public transportation, extended hours of operation, cleanliness and good service, food safety, and compliance with food regulations. Participants rated the importance of each factor on a Likert scale: “agree a lot” to “disagree a lot”.

The community food resources were categorized into five groups based on the literature [22,23]. Participants were asked if they shopped in the type of food stores listed within their neighborhood with “yes” or “no” options. For analyses, the community resources were regrouped as shown below, and composite scores were generated from participant’s responses for each community food resource category to evaluate the availability and accessibility of each resource category. The categories included:

1. Healthy food retail stores: This encompassed establishments such as supercenters, supermarkets, small grocery stores, ethnic stores, farmers’ or fruit and vegetable markets, and full-service restaurants;
2. Less healthy food retail stores: This included fast food restaurants, corner or convenience stores, convenience stores with gas stations, dollar stores, and vending machines;
3. Food assistance programs: This covered resources like food pantries or banks, community gardens, friend’s or relative places, churches or community centers, special supplemental nutrition programs for Women, Infants, and Children (WIC), and the Supplemental Nutrition Assistance Program (SNAP)/Food Stamps;
4. Food service resources: This category characterizes programs that basically assist families with ready-to-eat food including Meals on Wheels, National School Lunch Programs, School Breakfast Programs, and Summer Food Service Programs;
5. Mobile food resources: This category encompassed mobile food trucks, street food vendors, and food stands.

For analysis purposes, some variables responses were consolidated to reduce sparsity, improve model stability, and enhance results interpretation. For example, gender was recoded as man, woman, and others. Participants who indicated “yes” to more than one race were categorized as multi-race while single responses were retained.

The response options for motivators of shopping decisions were combined into 3-levels; “somewhat important” and “a little important” as “somewhat important”, “not applicable” and “not important at all” as “not important” and “very important”. For transportation mode, responses from participants who answered “yes” for more than one mode of transportation were first classified as multi-mode transportation. Then the single response was recoded as walk/bicycle, neighbor’s/friend’s car, public transportation, private car, and paid ride/home delivery. Lastly, the Likert scale response options for perceived community-level food-related needs were combined into 3-levels: “agree a lot” and “agree a little” as “agree”, “disagree a lot” and “disagree a little” as “disagree”, and “neither agree nor disagree”.

### 2.2. Statistical Approach

Descriptive statistics were reported using percentages for all categorical variables, mean ± standard deviation, as well as minimum and maximum values for all continuous variables. Using all the variables mentioned above in regression analyses (community resources as outcome variable and others as predictor variables), multicollinearity diagnostics were performed to detect highly correlated variables. This is to help mitigate the risk of unstable estimate and preventing overfitting in the models. The diagnostics identified a high correlation among the number of adults in the household, education, and food security status in all the regression models. Subsequently, the number of adults in the household and education were excluded from hierarchical regression analyses following. Figure S1 highlights the final variables that informed this study’s Anderson Behavioral Model. For the statistical analyses, the community food resources were the outcome variables, while the predictors variables included the predisposing, enabling, and need factors (the external environment was not included in the analyses).

The three blocks of predictors were sequentially entered into hierarchical regression models in this order predisposing, enabling, and need factors. Hence, three models were run for each community food resource category, model 1 included predisposing variables (i.e., age, marital status, number of children in a household, ethnicity, and race). Model 2 added enabling variables (i.e., income, travel time, travel mode, and motivators of shopping decisions). Model 3 (full model) added food security and perceived community-level food-related needs. This allowed for the testing of statistical significance with each block of predictors as well as the significance of individual predictors within blocks. We tested the collective predictive power of additional factors predicting the use of each community food resource category by reporting the adjusted R-squared. Statistical Analysis Software (version 9.4, 2016, SAS Institute, Inc., Cary, NC, USA) [24] was used and the level of significance consideration for the predisposing, enabling, and need factors was set at *p* < 0.05.

## 3. Results

### 3.1. Descriptive Statistics

A total of 1830 adults participated in the study. The sample’s age ranged from 18 to 79 with a mean age of 37.9  ±  12.1 (standard deviation, SD) years. The majority are White (87.6%), non-Hispanic (85.8%), and live in urban regions (56.6%). The participants reported an average travel time from their homes to food stores of 18.6 ± 18 SD minutes. The top 5 most frequently used community food stores include supermarkets (66%), fast food restaurants (64.3%), friends/relatives (53.2%), soup kitchens (33%), and street food vendors (36.5%). About half of the participants (50.4%) use multiple transportation modes to travel to get food and 66.7% reported being food insecure. Details on the background characteristics of the participants are described in Table S1; Summary of predisposing, enabling, need factors, and use of different community food resources among adults.

### 3.2. Hierarchical Regression Analysis

Results of the hierarchical regression are presented in Table 1, Table 2, Table 3, Table 4 and Table 5 for the community food resources. Table 1 shows the results of the hierarchical regression for healthy food retail stores. Model 1 shows that Hispanic and Asian were the significant predisposing factors; indicating that individuals identifying as Hispanic (β = −0.08, *p* = 0.04) were less likely to use healthy food retail stores and Asians were more likely to use healthy food retail stores compared to the Whites (β = 0.20, *p* = 0.01). In model 2, no predisposing factor was significant but travel time (β = −0.002, *p* = 0.04), multi-mode transportation (β = 0.14, *p* = 0.002), perceiving the ability to use WIC (β = −0.13, *p* = 0.02), and sales and coupons as “very important” in influencing shopping decision (β = 0.18, *p* = 0.01) were significantly enabling factors that were associated with the use of healthy food retail stores in the community. Within model 3, no predisposing factor was significant but traveling by private car and multi-mode transportation (β = 0.20, *p* < 0.001 and β = 0.14, *p* = 0.002, respectively) were the significant enabling factors. The significant need factors in model 3 included perceiving the ability to use of sales and coupons as “very important” in shopping decision (β = 0.17, *p* = 0.02), and food security (β = 0.16, *p* < 0.001). Model 3 (*F* = 2.42, *p* < 0.0001, Δ*R*^2^ = 0.11) has a higher proportion of variance in the predicting factors to explain the use of health food retail stores compared to model 1 and 2 (*F* = 1.49, *p* = 0.09, Δ*R*^2^ = 0.01; *F* = 2.63, *p* < 0.0001, Δ*R*^2^ = 0.08).

Table 2 shows the results for less healthy food retail stores. In model 1, age (β = −0.004, *p* = 0.01) and households with higher number of older children (β = 0.05, *p* = 0.02) were the predisposing factors associated with the use of less healthy retail stores. Age was the only predisposing factors that was significant in model 2 (β = −0.005, *p* = 0.02). The significant enabling factors in model 2 included using multi-mode transportation (β = 0.17, *p* < 0.001) and private cars (β = 0.17, *p* = 0.01). All predisposing and enabling factors that were significant in model 2 remained significant in model 3; (age: β = −0.005, *p* = 0.02; multi-mode transportation: β = 0.17, *p* = 0.01; and private cars: β = 0.17, *p* = 0.03). The significant need factors in model 3 included being neutral to perceiving low food price as a community-level food-related need (β = 0.11, *p* = 0.03). Model 3 explains 7% of the variance in the predicting factor for the use of less healthy food retail stores (*F* = 1.35, *p* = 0.05) while models 1 and 2 were *F* = 1.96, *p* = 0.02, Δ*R*^2^ = 0.02; *F* = 1.63, *p* = 0.02, Δ*R*^2^ = 0.05, respectively.

Table 3 shows the results for food assistance programs. In model 1, age (β = −0.02, *p* < 0.0001) and identifying as Asian (β = −0.64, *p* = 0.001) were the predisposing factors negatively associated with the utilization of food assistance programs. However, being married (β = 0.47, *p* < 0.001), Hispanic (β = 0.25, *p* = 0.02), households with a higher number of younger children (β = 0.30, *p* < 0.0001) and older children (β = 0.10, *p* = 0.02) were the predisposing factors positively associated with the utilization of food assistance programs. Model 2 shows that age and households with a higher number of younger children (β = 0.22, *p* = 0.001) were the significant predisposing factors. Multi-mode transportation (β = 0.46, *p* = < 0.001), perceiving the ability to use WIC (β = 0.97, *p* < 0.0001) and delivery option (β = 0.29, *p* = 0.02) in influencing shopping decisions, and household income between USD 50,000 and USD 99,999 (β = 0.46, *p* = 0.01) were the enabling factors associated with using a food assistance program in model 2. All the significant predisposing and enabling factors in model 2 except perceiving the ability to use delivery option in influencing shopping decision remained significant in model 3; indicating that households with a higher number of younger children (β = 0.23, *p* = 0.0002), multi-mode transportation (β = 0.49, *p* = < 0.001), perceiving the ability to use WIC in influencing shopping decisions (β = 0.80, *p* < 0.0001), and household income between USD 50,000 and USD 99,999 (β = 0.48, *p* = 0.01) were associated with using food assistance programs. The significant need factor in model 3 was food security (β = −0.29, *p* = 0.001). Model 1 explains a lower proportion of the variance in predicting factors for the use of less healthy food retail resources at 15% compared to models 2 and 3 at 36 and 37%, respectively (*F* = 19.30, *p* < 0.0001, Δ*R*^2^ = 0.15; *F* = 18.30, *p* < 0.0001, Δ*R*^2^ = 0.36; *F* = 11.48, *p* < 0.0001, Δ*R*^2^ = 0.37, respectively).

Table 4 shows the results for food service resources. Predisposing factors that were significant in model 1 include age (β = −0.03, *p* < 0.0001), being married (β = 0.69, *p* < 0.0001), households with a higher number of older children (β = 0.18, *p* < 0.0001) and younger children (β = 0.25, *p* = 0.0001), Hispanic (β = 0.37, *p* = 0.0001), Asians (β = −0.67, *p* = < 0.001), Blacks (β = −0.37, *p* = 0.01), and multi-race group (β = −0.51, *p* = 0.03). For model 2, the significant predisposing factors were age (β = −0.01, *p* = < 0.001), being married (β = 0.25, *p* = 0.03), households with a higher number of older children (β = 0.14, *p* < 0.001), younger children (β = 0.17, *p* = 0.002), Blacks (β = −0.48, *p* = 0.01), and Native Hawaiians (β = −1.32, *p* = 0.03). Travel time (β = 0.004, *p* = 0.03), multi-mode transportation (β = 0.58, *p* < 0.0001), perceiving the ability to use WIC (β = 0.70, *p* < 0.0001), and delivery option (β = 0.21, *p* = 0.04) as an influence on shopping decisions, and food price (β = 0.51, *p* < 0.001) were the significant enabling factors. With model 3, age (β = −0.01, *p* = 0.006), married (β = 0.24, *p* = 0.03), households with a higher number of older children (β = 0.13, *p* = 0.003) and younger children (β = 0.17, *p* = 0.003), and Blacks (β = −0.01, *p* = 0.006) were the significant predisposing factors. Multi-mode transportation (β = 0.57, p < 0.0001), perceiving the ability to use WIC as an influence on shopping decisions (β = 0.44, p = 0.0003) and food price (β = 0.45, p = 0.001) are also noteworthy enabling factors. On the other hand, food security emerges as a significant need factor (β = −0.52, p < 0.0001). Model 3 has a higher proportion of variance in the predicting factors to explain the use of food service resources compared to models 1 and 2 (*F* = 18.70, *p* < 0.0001, Δ*R*^2^ = 0.49; *F* = 28.26, *p* < 0.0001, Δ*R*^2^ = 0.47; *F* = 37.73, *p* < 0.0001, Δ*R*^2^ = 0.25, respectively).

Table 5 shows results for mobile food resources. Model 1 was statistically significant for age (β = −0.02, *p* < 0.0001), households with a higher number of younger children (β = 0.15, *p* = 0.0001), Hispanic (β = 0.30, *p* < 0.001), multi-race (β = −0.46, *p* = 0.01), and Asians (β = −0.49, *p* < 0.001). In model 2, only Native Hawaiians (β = −0.97, *p* = 0.04) was the significant predisposing factor. Using a neighbor’s or friend’s car (β = −0.46, *p* = 0.003), using multi-mode transportation (β = 0.30, *p* = 0.01), having a private car (β = −0.26, *p* = 0.02), household income between USD 50,000 and USD 99,999 (β = 0.13, *p* = 0.01), and perceiving the ability to use WIC as an influence on shopping decisions (β = 0.35, *p* < 0.0001) were the significant enabling factors. All the significant variables in model 2 remains significant in model 3 with the addition of food security as the significant need factor. This include having a private car (β = −0.41, *p* < 0.001), using a neighbor’s or friend’s car (β = −0.40, *p* = 0.01), multi-mode transportation (β = 0.17, *p* = 0.02), having a household income between USD 50,000 and USD 99,999 (β = 0.32, *p* = 0.01), perceiving the ability to use WIC as an influence on shopping decisions (β = 0.22, *p* = 0.02). Food security (β = −0.19, *p* = 0.02) and disagreeing with community-level need for culturally-relevant foods (β = −0.24, *p* = 0.02) were the significant need factors. Model 3 has a higher proportion of variance in the predicting factors to explain the use of mobile food resources compared to models 1 and 2, (*F* = 11.61, *p* < 0.0001, Δ*R*^2^ = 0.38; *F* = 28.97, *p* < 0.0001, Δ*R*^2^ = 0.20; *F* = 18.34, *p* < 0.0001, Δ*R*^2^ = 0.36, respectively).

## 4. Discussion

We adopted the Andersen Behavioral Model to examine the factors associated with the use of five different community food resources. The results from this study indicate that different factors predict how people use each category of food resources in their community.

For healthy food retail stores, compared to Whites, Asians are more likely to use a healthy food retail store. Although these predisposing factors were not prevalent in the second and full models, this finding adds to the discussion on racial/ethnic differences in diet and food demand. Gustavsen et al. [25] reported that non-Hispanic Asians consume the most seafood, fruit, and vegetables which are more accessible in retail store types within the healthy food retail category (including farmers markets, ethnic markets, supermarkets, and full-service restaurants). Also, this finding confirms previous research that reported a 1:1 ratio among Asians who shopped primarily in ethnic vs. non-ethnic grocery stores. We also found that Hispanics were less likely to use a healthy food retail store. This aligns with previous studies that reported that Hispanic shop more often at ethnic-focused stores, [26] dollar stores, drug stores, and discount grocers and less in regular grocery stores, and farmer’s markets [27]. Additionally, travel time multi-mode transportation and motivator of shopping decisions (using WIC benefits and coupons) were significant enabling factors to using a healthy food retail store. These factors, except travel time, remain significant with the addition of modes of transportation (private cars) and food security in the full model. These findings are consistent with previous studies that reported that transportation (especially time and cost) [28,29,30] are important for access to healthy food retail stores. The combination of these two factors confirms indicators of access to supermarkets in previous food access studies such as Food Environment and Food Access Research Atlases [31]. Coupons enhance marketing for retail outfits and are a cost-saving tool for consumers. A meta-analysis study reported that more than half of the American population uses coupons, saving more than USD 3 billion annually [32]. Therefore, coupons could be used as a tool to motivate shopping in a healthy food retail store. Moreover, the findings on food security confirm the association between food insecurity status and shopping behaviors [33]. A study among shoppers in small grocery stores found that people experiencing food insecurity are likely to shop for less healthy food items. Another study found a significant association between food insecurity and shopping in less healthy retail stores like dollar stores but null for stores like supermarkets [34]. Overall, to improve the demand for food in the healthy food retail stores, policies, and programs that would improve transportation access, encourage people to obtain and redeem food coupons for families experiencing food insecurity, and provide culturally relevant foods within this community food environment settings are needed.

For less healthy food retail stores, the use of less healthy retail stores decrease with age, and families with a higher number of older children display a tendency to engage with such food resources. Interestingly, these were prevalent in all the models. This is no surprise because fast-food consumption is prevalent among adolescents and younger adults [34]. Moreover, older adults are at greater risk of chronic diseases and may potentially have higher recognition of the health implications associated with the consumption of less nutritious foods [35]. Access to multi-mode transportation has the potential to enhance mobility; however, it may highlight infrastructure disparities for certain individuals. In such cases, it may indicate inconsistent access to a reliable transportation, thereby leading to an increase in the accessibility and utilization of unhealthy food resources, as observed in this present study [36]. Nevertheless, improved access to reliable transportation could potentially be directed toward facilitating access to healthier food stores. Multi-mode transportation, households with a higher number of children, and neutrality to food price considerations emerged as substantial factors in model 3. It is interesting that people who are ambivalent to needing lower food prices in their community are more likely use less healthy food retail stores. Their neutrality to food prices may make them more inclined to opt for less healthy food options available at a lower cost. However, food price/affordability remains a crucial aspect of public health that must be considered when addressing access to healthy food [37]. These results highlight relationships among age, household size, transportation access, and perceived need variables that influence people’s use of less healthy retail choices. This emphasizes how crucial it is to create tailored initiatives and programs that account for these multifaceted aspects to encourage healthier dietary choices and enhance well-being, particularly among younger adults. This may include nutrition education programs or subsidies/incentives that make healthier food options more affordable for individuals with neutral opinions on low food prices.

Regarding food assistance programs, this study consistently highlights households with a higher number of younger children as a significant predisposing factor and this is prevalent in all the models. Distinct food assistance programs such as WIC and SNAP have consistently relied on income and family size as primary eligibility criteria [38,39]. However, recent research on the correlation between program utilization, income, family composition, and structure are limited. Moreover, previous studies have indicated that the integration of food pantries with other assistance programs effectively addresses the unmet food needs of families with children [40,41]. Notably, our finding that the utilization of food assistance programs decrease with age in model 1 correlates with a previous study [42]. Older adults may be challenged by mobility constraints [43] and lack of awareness and information [44] to physically access food assistance programs. Our findings on race/ethnicity and use of food assistance programs align with a previous study that reported that Asians and Hispanic are among the races and ethnicity that least access SNAP [45,46] and will least likely use this type of food resource. It is evident that participation in food assistance programs could improve food insecurity among low-income populations [47,48]. However, improved access to reliable multiple means of transportation is important to encourage the use of food assistance programs among families in need and the elderly [28,49]. Moreover, some of these findings align with Wang et al.’s report on age, race, and food security as predicting factors for the use of informal food support [8]. Overall, the household size, modes of transportation, and use patterns highlight the need for specialized program approaches. Adopting a family-centric approach that addresses the challenges families face underscores the need for extending program reach, fostering greater participation, and enhancing benefits to ensure improved food access.

Being married, age, family composition, transportation, race, and ethnicity play a role in the use of food service resources, just like it was observed for food assistance programs. Consistent with the food assistance programs’ results, Hispanics were more likely to use food service resources. However, the likelihood of utilizing these resources tends to decline with age, while Asians and Blacks were less inclined to utilize this type of resource. Many eligible families already do not participate in or are on the waiting list for programs like the School Nutrition and Meals on Wheels [50,51]. Previous studies have also reported that race and ethnicity are salient factors that influence participation in and preference for meals served in these programs [52,53]. Furthermore, income and multi-mode of transportation were factors that contribute to the use of food services. This may be because clients in Meals on Wheels may have to cover a portion of their meal expenses though assistance comes from other funding sources. (e.g., Title III of the Older Americans Act, State general funds, Medicaid, charitable contributions) [54]. Similarly, children from families between 130 and 185 percent of the Federal poverty line can only receive a reduced-price lunch [55]. The observed association of longer travel time with use of food services could be that people using food service resources have unreliable transportation and already travel longer distance to get food [56]. Households that are food secure are less likely to use this resource, and this indicates that households with food needs may already be engaged in these services. However, more funding should be allocated so these programs can expand what they are able to offer to families in need, including cultural considerations for the type of meal served and improved distribution channels.

Mobile food resources are less likely to be accessed as the age advances and among Asians. In contrast, families with a larger number of younger children in the household show a positive association with an increased use of mobile food services. Given the priority of feeding children, the mobile food model has been previously used in the charitable food system by dispatching food trucks to low-income communities with large populations of young children [57]. This finding may also offer insights on the opportunity a mobile food resources can offer to older adults. This type of food resources may have great flexibility and considerable potentials to meet different dimensions of food access (including improving access to culturally-relevant foods) among low-income families that are already food insecure [58,59]. It is not surprising that individuals with inconsistent access to reliable transportation may be receptive to mobile food resources within their communities [60]. Furthermore, mobile produce markets are providing an emerging [54,59] response to improved fruit and vegetable access in underserved communities [61]. However, the correlations observed between the use of mobile food resources, income, Hispanics, and race variables are not clear. Nonetheless, the correlation between the use of mobile food resources and shopping using WIC may be explained by the potential for WIC participants to redeem their fruits and vegetable vouchers in a participating farm stand [62], including mobile produce markets [61]. In summary, mobile food services have the potential for improved access to healthy food options for families living in underserved communities. Thus, mobile food resources can be conduit for healthier food access among communities with unreliable transportation, families with children, and the elderly.

### Limitations

The results of this study should be interpreted in light of a few limitations. First, the study acknowledges the potential sampling bias inherent in individuals who choose to participate in MTurk studies. MTurk workers may not be fully representative of the broader population often exhibiting differences in demographics, socio-economic status, and other characteristics. We recognize the concern for an inconsistent answer that may ensue from online crowdsourcing platforms like MTurk and the researchers implemented robust quality control measures to eliminate invalid responses and adhered to recommended best practices to increase the validity and reliability of the participants responses [63]. Second, (self-)selection bias may also be an issue in this study, since adults who agree to participate in this study may initially be interested in nutrition and healthy food purchases. To mitigate this bias, prescreening questions in the study design allowed the researchers to filter out participants based on specific criteria and ensure that the sample aligns with the study desired characteristics. Third, the self-reported method used for data collection may be subject to recall and response biases. However, participants were assured anonymity and confidentiality of their responses in the study information page which may have limited response biases in the study. Additionally, the questions were framed in a neutral way and pretested to minimize potential biases. Fourthly, the cross-sectional design does not allow drawing any conclusions on the causal relationships between food environment/resources and predisposing, enabling, and need factors. Lastly, the Anderson model helped identify factors that influence food resource utilization and considers the potential for neighborhood self-selection (i.e., people choose neighborhoods based on their preferred facilities and resources). It may not explicitly address the dynamic interplay between utilization and those influencing factors in a way that directly accounts for reverse causality (e.g., the use of certain food resources can, in turn, affect a person’s predisposing or enabling factors). Despite these limitations, the study has significance in that standard tests of the instruments were conducted to establish its internal validity.

## 5. Conclusions

This study highlights significant racial/ethnic disparities in the use of healthy food retail stores, emphasizing the crucial role of factors such as travel time, transportation, and coupon use. Interventions aimed at improving transportation access and incentivize healthy shopping behaviors are recommended. For less healthy food retail stores, age-related trends and the impact of transportation on unhealthy food access are evident, emphasizing the need to address infrastructure disparities. Overall, comprehensive initiatives considering age, household size, transportation access, and food affordability are recommended to promote healthier dietary choices, particularly among younger adults. This may include introducing programs or subsidies that make healthier food options more affordable for individuals with neutral opinions on low food prices.

Furthermore, the study consistently identifies household characteristics, such as the number of younger children, age, race/ethnicity, and transportation access, as significant factors influencing the utilization of food assistance programs and services. It underscores the need for specialized program approaches, particularly for larger families and older adults facing mobility challenges. Additionally, addressing neighborhood-level access to nutritious foods is a crucial topic for consideration among older adults. To enhance participation in food assistance programs and encourage effective benefits use, adopting a family-centric approach, addressing cultural considerations, and allocating more funding for program expansion are recommended. Nonetheless, further research is needed to investigate the relationships between program utilization, income, family composition, and structure.

Moreover, the impact of having access to multi-mode transportation on food access can vary based on individual circumstances and local contexts. While it can enhance mobility and food choice for some, it may pose challenges related to costs and infrastructure disparities for others. Sustainable urban planning and policies can play a crucial role in maximizing the benefits of multi-mode transportation for improved food access among families in need and the elderly.

Mobile food resources hold significant promise in addressing various dimensions of food access, particularly among low-income families with children facing food insecurity, individuals with inconsistent access to reliable transportation and older adults. Mobile produce markets, as an emerging response, contribute to improved fruit and vegetable access in underserved communities. In essence, mobile food services can serve as vital channels for promoting healthier food access in particularly among individuals with mobility limitation, those that have appreciation for culturally-relevant foods and in communities with unreliable transportation.

In conclusion, it is important to design initiatives and strategies that would effectively transform food store and programs food offering, distribution logistics, and transportation access. This effort is crucial to enhance access to healthier community food resources for individuals and families facing dire need and residing in underserved areas.

## Figures and Tables

**Table 1:** Hierarchical regression analysis of predisposing, enabling, and need factors for use of healthy food retail stores.

Variables	Model 1 (R-square = 0.01)		Model 2 (R-square = 0.08)		Model 3 (R-square = 0.11)	
	ß	*p*	ß	*p*	ß	*p*
**Age**	0.00002	0.87	−0.002	0.35	−0.003	0.12
**Marital Status** ^1^						
married separated widowed	0.04 0.04 0.05	0.32 0.55 0.70	−0.01 0.09 −0.02	0.92 0.35 0.92	0.003 0.12 −0.02	0.95 0.24 0.90
**Household size** older children (5–17 years) younger children (<5 years)	0.0003 0.02	0.99 0.50	−0.01 0.04	0.57 0.16	−0.01 0.04	0.82 0.13
**Latinx** ^2^						
Hispanic	−0.08	**0.04**	−0.02	0.71	−0.003	0.95
**Race** ^3^						
American Indian Asian Black American Native Hawaiian multi-race	−0.11 0.20 −0.01 0.49 −0.09	0.50 **0.01** 0.90 0.09 0.35	−0.05 0.13 0.07 0.45 −0.06	0.77 0.14 0.36 0.12 0.60	−0.06 0.12 0.06 0.30 −0.07	0.74 0.18 0.46 0.29 0.60
**Travel Time**			−0.002	**0.04**	−0.001	0.13
**Travel Mode** ^4^						
bus friend’s/neighbor’s car paid ride/home delivery private car multi-mode			−0.15 0.12 −0.08 0.22 0.15	0.36 0.22 0.71 **<0.0001** **0.002**	−0.13 0.10 −0.09 0.20 0.15	0.44 0.31 0.64 **0.0004** **<0.002**
**Income** ^5^						
USD 25,000–49,999 USD 50,000–99,999 USD 100,000 and above			0.05 0.08 0.15	0.56 0.29 0.07	0.02 0.04 0.09	0.85 0.58 0.31
**Shopping Decision**						
Using WIC ^6^						
somewhat important very important			−0.10 −0.13	**0.04** **0.01**	−0.03 0.80	0.60 0.31
Using great sales and coupons ^6^						
somewhat important very important			0.10 0.18	0.13 **0.01**	0.09 0.17	0.19 **0.02**
Using price ^6^						
somewhat important very important			0.01 −0.02	0.90 0.76	0.003 −0.02	0.97 0.73
Using delivery services ^6^						
somewhat important very important			0.05 0.05	0.29 0.35	0.07 0.07	0.14 0.23
Food price ^7^ very inexpensive somewhat expensive very expensive			−0.01 0.02 −0.004	0.91 0.62 0.95	−0.01 0.03 −0.0003	0.95 0.39 1.00
**Food Security** ^8^						
food secure					0.16	**0.001**
**Perceived Residents Food-related Need** ^9^						
Improved selection					−0.03	0.57
neither agree nor disagree					0.01	0.94
disagree						
Great variety of foods					−0.09	0.06
neither agree nor disagree					−0.04	0.57
disagree						
More cultural relevant foods					−0.06	0.15
neither agree nor disagree					0.01	0.90
disagree						
Low prices of foods					0.01	0.75
neither agree nor disagree					−0.08	0.20
disagree						
Better quality of foods					0.03	0.50
neither agree nor disagree					−0.01	0.84
disagree						
Easy accessibility to public transportation					−0.03	0.48
neither agree nor disagree					0.05	0.44
disagree						
Improved convenient hours of operation					0.07	0.10
neither agree nor disagree					0.10	0.12
disagree						
Improved cleanliness and good service					−0.07	0.10
neither agree nor disagree					−0.06	0.37
disagree						
Good food safety practices					−0.01	0.82
neither agree nor disagree					0.07	0.35
disagree						

Model reference: ^1^ never married, ^2^ non-Hispanic, ^3^ White/Caucasian, ^4^ walk/bicycle, ^5^ below USD 25,000, ^6^ not at all important, ^7^ not expensive, ^8^ food insecure, ^9^ disagree. Statistically significant *p*-values are in bold.

**Table 2:** Hierarchical regression analysis of predisposing, enabling, and need factors for use of less healthy food retail stores among adults (N = 1830).

Variables	Model 1 (R-square = 0.02)		Model 2 (R-square = 0.05)		Model 3 (R-square = 0.07)	
	ß	*p*	ß	*p*	ß	*p*
**Age**	−0.004	**0.01**	−0.005	**0.02**	−0.005	**0.02**
**Marital Status** ^1^						
married separated widowed	0.03 0.06 −0.14	0.60 0.45 0.38	0.05 0.11 −0.11	0.44 0.40 0.63	0.06 0.12 −0.08	0.40 0.34 0.73
**Household size** older children (5–17 years) younger children (<5 years)	0.05 0.01	**0.02** 0.66	0.04 −0.01	0.09 0.87	0.05 −0.01	0.07 0.82
**Latinx** ^2^						
Hispanic	−0.03	0.52	0.01	0.92	0.02	0.73
**Race** ^3^						
American Indian Asian Black American Native Hawaiian multi-race	−0.28 0.0001 0.01 −0.11 −0.08	0.14 1.00 0.88 0.76 0.83	−0.31 0.01 −0.01 −0.02 0.05	0.13 0.93 0.93 0.95 0.74	−0.30 −0.003 −0.01 −0.08 0.04	0.16 0.98 0.90 0.82 0.80
**Travel Time**			0.001	0.48	0.001	0.47
**Travel Mode** ^4^						
bus friend’s/neighbor’s car paid ride/home delivery private car multi-mode			−0.20 0.06 −0.20 0.17 0.17	0.34 0.62 0.43 **0.01** **<0.01**	−0.20 0.07 −0.22 0.16 0.17	0.32 0.59 0.40 **0.03** **0.01**
**Income** ^5^						
USD 25,000–49,999 USD 50,000–99,999 USD 100,000 and above			0.01 0.14 0.02	0.10 0.10 0.11	−0.03 0.10 −0.02	0.79 0.31 0.86
**Shopping Decision**						
Using WIC ^6^						
somewhat important very important			0.003 −0.02	0.97 0.72	0.01 −0.02	0.90 0.82
Using great sales and coupons ^6^						
somewhat important very important			0.13 0.13	0.14 0.12	0.10 0.12	0.24 0.18
Using price ^6^						
somewhat important very important			−0.06 −0.04	0.47 0.64	−0.07 −0.04	0.43 0.64
Using delivery services ^6^						
somewhat important very important			0.03 0.05	0.63 0.47	0.02 0.05	0.76 0.51
Food price ^7^ very inexpensive somewhat expensive very expensive			0.03 0.02 0.02	0.69 0.76 0.82	0.02 0.02 0.02	0.82 0.72 0.83
**Food Security** ^8^						
food secure					0.06	0.30
**Perceived Residents Food-related Need** ^9^						
Improved Selection					−0.03	0.61
neither agree nor disagree					0.08	0.43
disagree						
Great variety of foods					−0.01	0.90
neither agree nor disagree					−0.39	0.69
disagree						
More cultural relevant foods					0.03	0.60
neither agree nor disagree					−0.02	0.79
disagree						
Low prices of foods					0.12	**0.03**
neither agree nor disagree					−0.08	0.35
disagree						
Better quality of foods					0.01	0.93
neither agree nor disagree					0.02	0.83
disagree						
Easy accessibility to public transportation					0.05	0.34
neither agree nor disagree					0.003	0.97
disagree						
Improved convenient hours of operation						
neither agree nor disagree					0.001	0.99
disagree					−0.13	0.11
Improved cleanliness and good service						
neither agree nor disagree					−0.04	0.50
disagree					−0.05	0.54
Good food safety practices						
neither agree nor disagree					−0.18	0.75
disagree					0.14	0.12

Model reference: ^1^ never married, ^2^ non-Hispanic, ^3^ White/Caucasian, ^4^ walk/bicycle, ^5^ below USD 25,000, ^6^ not at all important, ^7^ not expensive, ^8^ food insecure, ^9^ disagree. Statistically significant *p*-values are in bold.

**Table 3:** Hierarchical regression analysis of predisposing, enabling, and need factors for use of food assistance programs among adults (N = 1830).

Variables	Model 1 (R-square = 0.14)		Model 2 (R-square = 0.36)		Model 3 (R-square = 0.37)	
	ß	*p*	ß	*p*	ß	*p*
**Age**	−0.02	**<0.0001**	0.001	**0.02**	0.003	0.36
**Marital Status** ^1^						
married separated widowed	0.47 0.26 0.01	**<0.0001** 0.14 0.97	0.02 −0.13 −0.22	0.83 0.57 0.59	0.01 −0.16 −0.23	0.91 0.49 0.58
**Household size** older children (5–17 years) younger children (<5 years)	0.10 0.30	**<0.001** **<0.0001**	0.09 0.22	0.07 **0.0006**	0.08 0.23	0.10 **0.002**
**Latinx** ^2^						
Hispanic	0.25	**0.02**	−0.14	0.19	−0.16	0.12
**Race** ^3^						
American Indian Asian Black American Native Hawaiian multi-race	−0.18 −0.64 −0.13 −0.63 −0.40	0.67 **0.001** 0.40 0.11 0.39	−0.21 −0.20 −0.11 −0.64 0.33	0.58 0.32 0.55 0.33 0.24	−0.11 −0.16 −0.10 −0.58 0.40	0.77 0.45 0.61 0.39 0.16
**Travel Time**			0.002	0.30	0.001	0.55
**Travel Mode** ^4^						
bus friend’s/neighbor’s car paid ride/home delivery private car multi-mode			0.41 −0.31 −0.46 −0.20 0.47	0.28 0.16 0.32 0.11 **<0.0001**	0.43 −0.23 −0.34 −0.13 0.49	0.26 0.32 0.47 0.32 **<0.0001**
**Income** ^5^						
USD 25,000–49,999 USD 50,000–99,999 USD 100,000 and above			0.27 0.46 0.26	0.15 **0.01** 0.19	0.25 0.48 0.28	0.19 **0.01** 0.16
**Shopping Decision**						
Using WIC ^6^						
somewhat important very important			0.96 0.97	**<0.001** **<0.001**	0.79 0.80	**<0.0001** **<0.0001**
Using great sales and coupons ^6^						
somewhat important very important			0.05 −0.04	0.75 0.78	0.07 −0.04	0.66 0.82
Using price ^6^						
somewhat important very important			0.15 0.18	0.36 0.25	0.21 0.25	0.19 0.13
Using delivery services ^6^						
somewhat important very important			0.13 0.29	0.26 **0.02**	0.04 0.20	0.76 0.13
Food price ^7^ very inexpensive somewhat expensive very expensive			0.26 0.15 0.19	0.16 0.09 0.18	0.23 0.12 0.17	0.22 0.18 0.25
**Food Security** ^8^						
food secure					−0.29	**0.01**
**Perceived Residents Food-related Need** ^9^						
Improved Selection						
neither agree nor disagree					−0.12	0.26
disagree					−0.06	0.75
Great variety of foods						
neither agree nor disagree					0.03	0.78
disagree					−0.01	0.95
More cultural relevant foods						
neither agree nor disagree					−0.01	0.93
disagree					−0.11	0.42
Low prices of foods						
neither agree nor disagree					0.17	0.09
disagree					0.03	0.84
Better quality of foods						
neither agree nor disagree					−0.01	0.97
disagree					0.06	0.75
Easy accessibility to public transportation						
neither agree nor disagree					−0.08	0.38
disagree					0.06	0.64
Improved convenient hours of operation						
neither agree nor disagree					0.03	0.76
disagree					−0.16	0.26
Improved cleanliness and good service						
neither agree nor disagree					−0.02	0.83
disagree					0.02	0.89
Good food safety practices						
neither agree nor disagree					−0.09	0.41
disagree					−0.04	0.83

Model Reference: ^1^ never married, ^2^ non-Hispanic, ^3^ White/Caucasian, ^4^ walk/bicycle, ^5^ below USD 25,000, ^6^ not at all important, ^7^ not expensive, ^8^ food insecure, ^9^ disagree. Statistically significant *p*-values are in bold.

**Table 4:** Hierarchical regression analysis of predisposing, enabling, and need factors for use of food service resources among adults (N = 1830).

Variables	Model 1 (R-square = 0.25)		Model 2 (R-square = 0.47)		Model 3 (R-square = 0.49)	
	ß	*p*	ß	*p*	ß	*p*
**Age**	−0.03	**<0.0001**	−0.01	**0.0002**	−0.01	**0.01**
**Marital Status** ^1^						
married separated widowed	0.69 0.20 0.26	**<0.0001** 0.22 0.37	0.25 −0.04 0.28	**0.03** 0.87 0.44	0.24 −0.09 0.26	**0.03** 0.67 0.48
**Household size** older children (5–17 years) younger children (<5 years)	0.18 0.25	**<0.0001** **<0.0001**	0.14 0.17	**0.001** **0.003**	0.13 0.17	**0.003** **0.003**
**Latinx** ^2^						
Hispanic	0.37	**0.0001**	0.11	0.24	0.09	0.33
**Race** ^3^						
American Indian Asian Black American Native Hawaiian multi-race	−0.39 −0.67 −0.38 −1.17 −0.51	0.30 **0.003** **0.01** 0.09 **0.03**	−0.21 −0.21 −0.49 −1.32 0.08	0.54 0.26 **0.004** **0.03** 0.75	−0.17 −0.10 −0.43 −1.09 0.18	0.62 0.59 **0.01** 0.07 0.48
**Travel Time**			0.003	0.02	0.002	0.17
**Travel Mode** ^4^						
bus friend’s/neighbor’s car paid ride/home delivery private car multi-mode			0.09 −0.16 −0.46 −0.16 0.57	0.80 0.43 0.33 0.16 **<0.0001**	0.06 −0.06 −0.07 −0.07 0.57	0.87 0.78 0.86 0.53 **<0.0001**
**Income** ^5^						
USD 25,000–49,999 USD 50,000–99,999 USD 100,000 and above			0.11 0.22 −0.07	0.50 0.18 0.69	0.11 0.26 0.002	0.51 0.11 0.99
**Shopping Decision**						
Using WIC ^6^						
somewhat important very important			0.75 0.70	**<0.001** **<0.001**	0.49 0.44	**<0.0001** **0.0003**
Using great sales and coupons ^6^						
somewhat important very important			0.05 −0.14	0.75 0.32	0.08 −0.10	0.59 0.45
Using price ^6^						
somewhat important very important			0.02 −0.09	0.87 0.52	0.12 −0.01	0.42 0.97
Using delivery services ^6^						
somewhat important very important			0.21 0.21	**0.04** 0.06	0.11 0.12	0.32 0.29
Food price ^7^ very inexpensive somewhat expensive very expensive			0.26 0.15 0.19	0.16 0.09 0.18	0.12 0.20 0.45	0.47 **0.01** **0.001**
**Food Security** ^8^						
food secure					−0.52	**<0.0001**
**Perceived Residents Food-related Need** ^9^						
Improved Selection						
neither agree nor disagree					−0.04	0.71
disagree					−0.08	0.63
Great variety of foods						
neither agree nor disagree					0.03	0.75
disagree					0.04	0.78
More cultural relevant foods						
neither agree nor disagree					0.08	0.36
disagree					−0.06	0.62
Low prices of foods						
neither agree nor disagree					0.17	0.09
disagree					0.03	0.84
Better quality of foods						
neither agree nor disagree					0.18	0.05
disagree					0.12	0.40
Easy accessibility to public transportation						
neither agree nor disagree					−0.11	0.21
disagree					−0.18	0.13
Improved convenient hours of operation						
neither agree nor disagree					0.04	0.62
disagree					−0.11	0.37
Improved cleanliness and good service ^9^						
neither agree nor disagree					−0.0002	1.00
disagree					−0.23	0.13
Good food safety practices						
neither agree nor disagree					0.02	0.80
disagree					0.24	0.09

Model reference: ^1^ never married, ^2^ non-Hispanic, ^3^ White/Caucasian, ^4^ walk/bicycle, ^5^ below USD 25,000, ^6^ not at all important, ^7^ not expensive, ^8^ food insecure, ^9^ disagree. Statistically significant *p*-values are in bold.

**Table 5:** Hierarchical regression analysis of predisposing, enabling, and need factors for use of mobile food resources among adults (N = 1830).

Variables	Model 1 (R-square = 0.20)		Model 2 (R-square = 0.36)		Model 3 (R-square = 0.38)	
	ß	*p*	ß	*p*	ß	*p*
**Age**	1.14	**<0.0001**	−0.004	0.15	−0.002	0.51
**Marital Status** ^1^						
married separated widowed	0.47 0.03 0.07	**<0.0001** **<0.0001** 0.75	0.12 −0.12 −0.17	0.18 0.46 0.55	0.11 −0.18 −0.13	0.23 0.28 0.65
**Household size** older children (5–17 years) younger children (<5 years)	0.05 0.15	0.06 **0.0001**	0.04 0.07	0.18 0.09	0.04 0.08	0.21 0.07
**Latinx** ^2^						
Hispanic	0.30	**<0.0001**	0.08	0.26	0.06	0.38
**Race** ^3^						
American Indian Asian Black American Native Hawaiian multi-race	−0.24 −0.49 −0.16 −0.88 −0.45	0.40 **0.003** 0.11 0.08 **0.01**	−0.21 −0.27 −0.06 −0.96 −0.22	0.44 0.06 0.67 **0.04** 0.27	−0.19 −0.26 −0.03 −0.88 −0.19	0.48 0.09 0.83 0.06 0.34
**Travel time**			0.0003	0.83	−0.00004	0.98
**Travel Mode** ^4^						
bus friend’s/neighbor’s car paid ride/home delivery private car multi-mode			−0.38 −0.46 −0.54 −0.45 0.17	0.15 **0.003** **0.1** **<0.0001** **0.003**	−0.40 −0.40 −0.51 −0.41 0.17	0.14 **0.01** 0.12 **<0.0001** **0.03**
**Income** ^5^						
USD 25,000–49,999 USD 50,000–99,999 USD 100,000 and above			0.14 0.31 0.21	0.28 **0.02** 0.12	0.13 0.32 0.24	0.34 **0.01** 0.09
**Shopping Decision**						
Using WIC ^6^						
somewhat important very important			0.49 0.35	**<0.001** **<0.001**	0.36 0.22	**<0.0001** **0.02**
Using great sales and coupons ^6^						
somewhat important very important			0.22 0.13	0.05 0.26	0.20 0.11	0.07 0.34
Using price ^6^						
somewhat important very important			−0.07 −0.08	0.52 0.48	−0.04 −0.05	0.70 0.65
Using delivery services ^6^						
somewhat important very important			0.11 0.15	0.17 0.08	0.05 0.11	0.52 0.24
Food price ^7^ very inexpensive somewhat expensive very expensive			0.14 0.03 0.16	0.29 0.64 0.12	0.12 0.01 0.16	0.34 0.93 0.13
**Food Security** ^8^						
food secure					−0.19	**0.02**
**Perceived Residents Food-related Need** ^9^						
Improved Selection						
neither agree nor disagree					−0.01	0.89
disagree					−0.01	0.95
Great variety of foods						
neither agree nor disagree					0.05	0.52
disagree					0.07	0.56
More cultural relevant foods						
neither agree nor disagree					−0.03	0.60
disagree					−0.24	**0.02**
Low prices of foods						
neither agree nor disagree					0.13	0.05
disagree					0.08	0.49
Better quality of foods						
neither agree nor disagree					0.03	0.69
disagree					0.05	0.67
Easy accessibility to public transportation						
neither agree nor disagree					0.01	0.84
disagree					−0.05	0.58
Improved convenient hours of operation						
neither agree nor disagree					−0.04	0.60
disagree					0.10	0.32
Improved cleanliness and good service						
neither agree nor disagree					0.01	0.93
disagree					0.07	0.56
Good food safety practices						
neither agree nor disagree					−0.03	0.68
disagree					−0.06	0.61

Model reference: ^1^ never married, ^2^ non-Hispanic, ^3^ White/Caucasian, ^4^ walk/bicycle, ^5^ below USD 25,000, ^6^ not at all important, ^7^ not expensive, ^8^ food insecure, ^9^ disagree. Statistically significant *p*-values are in bold.

## Data Availability

Due to privacy and ethical restrictions, the dataset for this study will only be made available upon request.

## Supplementary Materials

The following supporting information can be downloaded at: https://www.mdpi.com/article/10.3390/ijerph21010076/s1, Figure S1: Anderson Behavioral Model Adapted for Use of Community Food Resources; Table S1: Summary of Predisposing, Enabling, Need Factors and Use of Different Community Food Resources among Adults.
