# Supplementary material for: Examining Factors Associated with the Use of Community Food Resources: An Application of the Andersen Model to Inform Future Interventions
Source: Int J Environ Res Public Health. 2024 Jan 9;21(1):76. doi: 10.3390/ijerph21010076 (PMC10815130; doi:10.3390/ijerph21010076)
Supplement: Supplementary file 1 [file ijerph-21-00076-s001.zip › ijerph-2702774-supplementary.pdf]

Figure S1. Anderson Behavioral Model Adapted for Use of Community Food Resources

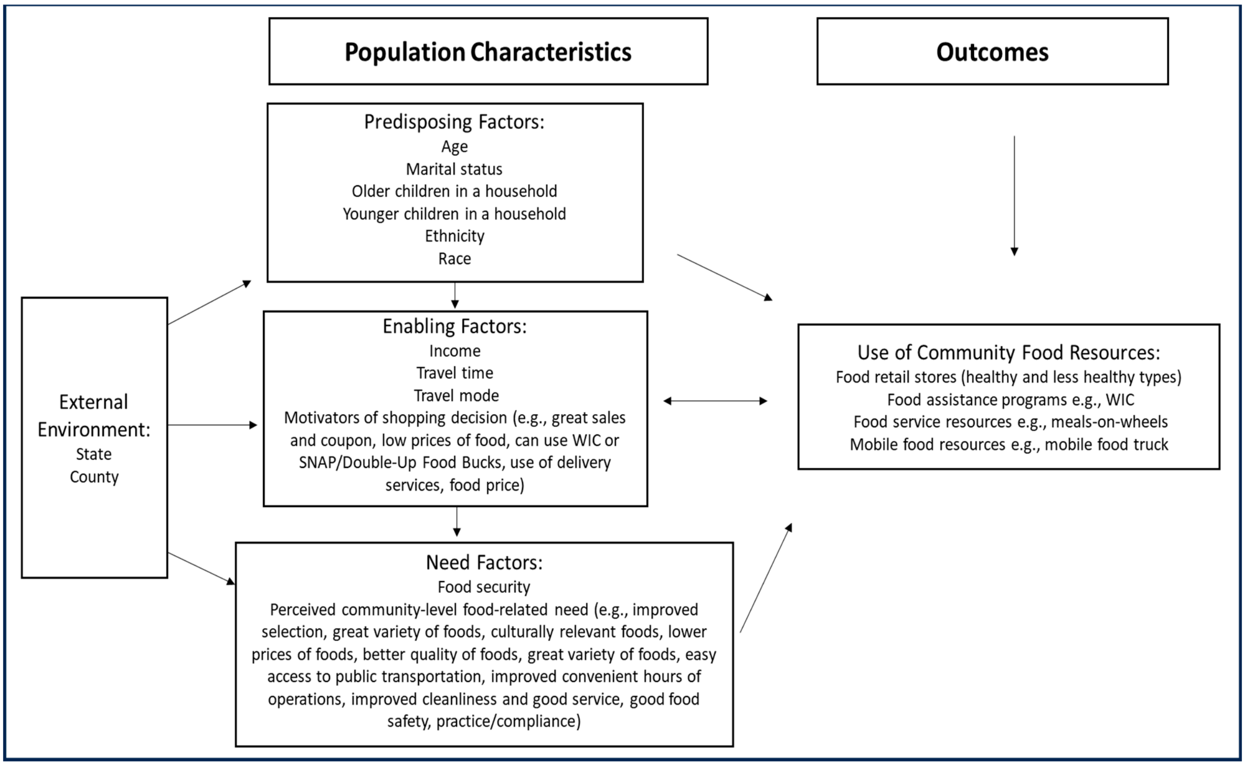

Table S1. Summary of Predisposing, Enabling, Need Factors, and Use of Different Community Food Resources among Adults (N=1830)

Variables	Mean (SD)	Min-Max	%
**Predisposing Factors**			
**Age (years)**	37.9 (12.1)	18-79	
**Gender**			
man			41.0
woman			57.8
others			1.2
**Education**			
up to high school			5.2
up to college degree			18.0
advanced degree			76.8
**Marital Status**			
married			74.2
separated			6.3
widowed			1.2
never married			18.3
**Number of Adults in the Household (Hhd)**			
1			5.8
2			20.0
3			50.7
4			14.4
more than 5			9.1
**Number of Older Children in the Hhd (5-17years)**			
0			46.3
1			38.2

2		12.9
3		1.7
more than 4		0.9
**Number of Younger Children in the Hhd (<5 years)**		
0		56.5
1		36.7
2		6.3
more than 3		0.5
**Ethnicity**		
Hispanic		14.2
non-Hispanic		85.8
**Race**		
White/caucasian		87.6
Black/African American		6.1
Asian		3.1
Native Hawaiian/other pacific		0.2
American Indian		0.8
multi-race		2.3
**Enabling Factors**		
**Travel Time (Minutes)**	18.6 (18)	
**Mode of Transportation**		
walk/bicycle		5.5
bus		1.0
private car		37.4
friend/neighbor's car		5.0
paid ride/home delivery		0.7
multi-mode		50.4
**Neighborhood Type**		
urban		56.6
sub-urban		34.7
rural		16.7
**Income**		
below $25,000		5.2
$25,000-$49,999		27.7
$50,000-$99,999		48.1
$100,000 and above		19.0
**Shopping Decisions**		
**Great sales and coupons**		
not at all important		5.8
somewhat important		50.6
very important		43.6
**Low price of food**		
not at all important		5.1
somewhat important		45.5
very important		49.4
**Could use of WIC or SNAP/Double-Up Food Bucks**		
not at all important		37.7
somewhat important		38.9
very important		23.4
**Could use delivery devices**		
not at all important		27.6
somewhat important		44.9
very important		27.5
**Rating of food prices**		
very inexpensive		4.2
not expensive		26.5
		59.4

somewhat expensive			
**Need Factors**			
**Perceived Residents' Food-related Need**			
**Improved selection**			
agreed			72.0
neither agree nor disagree			17.5
disagree			10.5
**Great variety of foods**			
agreed			68.6
neither agree nor disagree			19.6
disagree			11.8
**More cultural relevant foods**			
agreed			59.1
neither agree nor disagree			25.4
disagree			15.5
**Lower prices of foods**			
agreed			74.4
neither agree nor disagree			18.9
disagree			6.7
**Better quality foods**			
agreed			68.5
neither agree nor disagree			21.2
disagree			10.3
**Easier access to public transportation and bus-stops**			
agreed			52.9
neither agree nor disagree			28.8
disagree			18.3
**Improved convenient hours of operation**			
agreed			51.9
neither agree nor disagree			27.7
disagree			20.4
**Improved cleanliness and good service**			
agreed			55.2
neither agree nor disagree			26.5
disagree			18.4
**Good food safety practice/compliance**			
agreed			56.8
neither agree nor disagree			27.2
disagree			16.0
**Use of Community Resources**			
**Healthy Food Stores**			
supercenters			55.0
supermarkets			66.0
small grocery stores			41.0
ethnic stores			10.7
farmers'/fruits & vegetable market			16.0
healthy food retail composite score	0.52 (0.50)	0-1	
**Less Healthy Food Stores**			
fast food restaurant			64.3
corner or convenient stores			18.6
convenience stores with gas station			12.9
dollar store			13.5
vending machines			12.5
less healthy food stores composite score	0.77 (0.62)	0-2	

**Food Assistance Program**			
food pantry or bank			33.1
community garden			33.7
friend's/relative's place			53.2
church or community centers			14.7
Special Supplemental Nutrition Program for Women, Infants, and Children (WIC)			26.4
Supplemental Nutrition Assistance Program (SNAP)/food stamps			34.5
food assistance composite score	1.96 (1.38)	0-6	
**Food Services Resources**			
soup kitchen			33.0
meals-on-the-wheels			26.6
national school lunch program			23.7
school breakfast program			19.7
summer food service program			17.2
food services composite score	1.20 (1.38)	0-1	
**Mobile Food Services Resources**			
mobile food truck			32.2
street food vendors			36.5
food stand			23.9
mobile food services composite score	0.93 (0.98)	0	
